# Randomised prior feedback modulates neural signals of outcome monitoring

**DOI:** 10.1016/j.neuroimage.2015.10.046

**Published:** 2016-01-15

**Authors:** Faisal Mushtaq, Richard M. Wilkie, Mark A. Mon-Williams, Alexandre Schaefer

**Affiliations:** a School of Psychology, University of Leeds, Leeds, West Yorkshire, UK; b School of Business, Monash University, Sunway Campus, Selangor, Malaysia

**Keywords:** Feedback-related negativity, Reward prediction error, Short-term expectations, Salience, Monitoring, Uncertainty, Affective priming

## Abstract

Substantial evidence indicates that decision outcomes are typically evaluated relative to expectations learned from relatively long sequences of previous outcomes. This mechanism is thought to play a key role in general learning and adaptation processes but relatively little is known about the determinants of outcome evaluation when the capacity to learn from series of prior events is difficult or impossible. To investigate this issue, we examined how the feedback-related negativity (FRN) is modulated by information briefly presented before outcome evaluation. The FRN is a brain potential time-locked to the delivery of decision feedback and it is widely thought to be sensitive to prior expectations. We conducted a multi-trial gambling task in which outcomes at each trial were fully randomised to minimise the capacity to learn from long sequences of prior outcomes. Event-related potentials for outcomes (Win/Loss) in the current trial (Outcome_t_) were separated according to the type of outcomes that occurred in the preceding two trials (Outcome_t-1_ and Outcome_t-2_). We found that FRN voltage was more positive during the processing of win feedback when it was preceded by wins at Outcome_t-1_ compared to win feedback preceded by losses at Outcome_t-1_. However, no influence of preceding outcomes was found on FRN activity relative to the processing of loss feedback. We also found no effects of Outcome_t-2_ on FRN amplitude relative to current feedback. Additional analyses indicated that this effect was largest for trials in which participants selected a decision different to the gamble chosen in the previous trial. These findings are inconsistent with models that solely relate the FRN to prediction error computation. Instead, our results suggest that if stable predictions about future events are weak or non-existent, then outcome processing can be determined by affective systems. More specifically, our results indicate that the FRN is likely to reflect the activity of positive affective systems in these contexts. Importantly, our findings indicate that a multifactorial explanation of the nature of the FRN is necessary and such an account must incorporate affective and motivational factors in outcome processing.

## Highlights

• FRN is sensitive to prior events but does this persist when learning is impossible?
• Four FRN theories were examined in a task with fully randomised feedback.
• Previous trial outcome modulated FRN during positive but not negative outcomes.
• Results consistent with a positive affective modulation interpretation of the FRN.
• FRN is determined by factors beyond prediction error (e.g. affective/motivational).

## Introduction

The ability to rapidly update information about reward probability is necessary for goal-directed behaviour. Monitoring and evaluating an outcome relative to prior expectations is essential to this process (Kerns et al., 2004, Schall et al., 2002, Sohn et al., 2007). A large body of research on outcome monitoring has focused on a scalp event-related potential known as the feedback-related negativity (FRN; Gehring and Willoughby, 2002, Miltner et al., 1997). The FRN is usually operationalised as a contrast between ERPs to negative and positive outcomes. It has a frontocentral topography and is characterised by a negative deflection maximal at ~ 250–350 ms after feedback onset that is larger for non-reward compared to reward outcomes (Ferdinand et al., 2012, Yeung and Sanfey, 2004). Substantial evidence indicates that the FRN is linked to activity in medial frontal areas including the ACC (e.g. Hauser et al., 2014). The FRN is influenced by outcome history and varies as a function of prior reward expectations and probability: In fact, the FRN produced in the majority of previous studies relates to information that relies on learning contexts established over multiple trials and blocks (Bellebaum et al., 2010a, Cohen and Ranganath, 2007, Donkers et al., 2005, Pfabigan et al., 2010, Pietschmann et al., 2011, Sailer et al., 2010, Santesso et al., 2008).

A few studies have shown that the influence of prior outcome history on the FRN can be observed on very brief time scales. Specifically, information presented in the trial immediately preceding a current trial (Outcome_t-1_) can modulate the FRN related to the current trial (Outcome_t_) (e.g. Gehring and Willoughby, 2002, Goyer et al., 2008, Holroyd and Coles, 2002). These findings suggest that the FRN is sensitive to factors beyond the learning of probabilistic relationships between events and outcomes over a long period of time. These results are of importance to the field of decision neuroscience as they suggest that expectations can be formed rapidly and (ultimately) bias decision-making on a very short time scale. Whilst the determinants of FRN effects have been the focus of intense debates in recent years, the processes that modulate short-term effects on the FRN have received relatively little attention (we refer to these effects as the stFRN hereafter). Examining the stFRN promises not only to shed light on the debate about the determinants of the FRN, but also speaks to the broader issue of how the brain keeps track of changing expectancies in a rapidly-changing environment. Thus, the goal of this study is to test four contrasting explanations of stFRN encoding effects derived from existing FRN models.

First, the most prevalent account of the FRN has been provided by the reinforcement learning error-related negativity theory (RL-ERN; Holroyd and Coles, 2002). The original version of the RL-ERN theory proposed that the FRN indexes negative reward prediction error (-RPE) — a key component of Reinforcement Learning theories (e.g. Sutton and Barto, 1998). A -RPE occurs when an event (e.g. a decision outcome) violates a prediction learned from previous outcomes in such a way that the event constitutes an outcome that is “worse than expected”. More specifically, the RL-ERN theory posited that dopaminergic systems in mesencephalic areas monitor and detect when learned predictions are violated. When a -RPE is detected, there is a decrease in dopaminergic firing rate. This change in dopamine activity produces a signal that is sent to the ACC, causing a disinhibition of ACC neurons and thus leads to a larger FRN (Holroyd and Coles, 2002). These prediction error signals are suggested to be signals that trigger the implementation of top-down cognitive control processes (Kerns et al., 2004, Mushtaq et al., 2011, Swainson et al., 2003).

Second, a number of studies have reported a greater FRN not only for -RPE, but also for positive reward prediction errors (+RPE, e.g. Ferdinand et al., 2012, Oliveira et al., 2007). These findings contradict the RL-ERN model and support current models that emphasise a valence-independent explanation of the FRN. For instance, these results are consistent with accounts such as the predicted response-outcome (PRO) model (Alexander and Brown, 2011). The PRO model posits that ACC neurons keep track of the history of previous positive and negative reinforcements to specific actions, and formulate predictions about the probabilities of future outcomes. When a predicted outcome does not occur — a surprise — then ACC activity is maximal. Importantly, according to the PRO model, surprising outcomes are processed by ACC neurons in response to both reward and non-reward. This leads to the prediction that the FRN should index prediction errors irrespective of the sign of the error — as observed by Ferdinand et al. (2012) and Oliveira et al. (2007). Similarly, Talmi et al. (2013) recently suggested that the FRN codes salience prediction errors irrespective of outcome valence.

Beyond the original RL-ERN and valence-independent accounts, an interesting development in FRN research comes from the growing evidence showing that the FRN seems to be driven mainly by sensitivity to positive outcomes. A recent review of the literature (Walsh and Anderson, 2012) reported that a number of FRN studies tend to show that the negativity of the component is attenuated for outcomes that are better than expected. This effect results in a greater positivity for +RPEs, whereas the FRN waveform related to negative outcomes often remains a clear negative peak that varies little (or not at all) as a function of -RPE. This contrast between a varying FRN positivity to +RPEs and a relatively stable FRN to -RPEs would, in many cases, be sufficient to account for the classic FRN component. Walsh and Anderson (2012) noted that this predominance of FRN positivity is present in a majority of FRN studies, whereas experiments reporting an increased negativity for -RPEs are less frequent. This trend in the literature has led to a re-formulation of the RL-ERN model by its original authors, who more recently proposed that the FRN observed on the scalp is the product of two distinct outcome-monitoring processes (Holroyd et al., 2008). Specifically, the revised account holds that both phasic increases and decreases modulate the FRN. A negative deflection (N2) is produced by low probability (i.e. unexpected) task relevant events, irrespective of valence. However, unexpected rewards also produce a positive deflection induced by a phasic increase in dopaminergic activity — referred to as the “reward positivity” (Baker and Holroyd, 2011, Holroyd and Yeung, 2012, Holroyd et al., 2011). This increase in dopamine firing rate inhibits ACC neurons, thus causing a reduction in the N2-like negativity typical of the FRN. This model fits with the majority of the data reviewed by Walsh and Anderson (2012), and has received further recent support (Holroyd and Yeung, 2012).

Nevertheless, data exist that do not seem to be explained by the updated RL-ERN model (we refer to this as reward positivity [RP] model from hereon in). Apart from evidence supporting valence-independent accounts, there are also studies reporting a more positive FRN amplitude when positive outcomes are expected rather than unexpected. For instance, in a gambling task, San Martín et al. (2010) found that the FRN was more positive for “win” outcomes when the probability of rewards was higher, compared to win outcomes in a low reward probability context. Similarly, Mushtaq et al. (2013), in a different decision task, found greater FRN positivity for “win” outcomes in a context of positive compared to negative expectations. In related findings, Yu and Zhang (2014) did not find a greater FRN positivity for rewards compared to non-rewards when losses were more likely- contradicting a key prediction of the reward positivity framework. In addition, they found a more positive FRN for reward (compared to non-reward) outcomes in the context of positive expectations.

These studies point towards a fourth account of the FRN. It is possible that, in the studies mentioned above, a positive context (e.g. a “gain” domain, or reward expectation) could have primed a positive affect system. In other words, a positive context may have pre-activated affective systems sensitive to appetitive stimuli, which in turn became more sensitive to the delivery of reward feedback. This possibility is consistent with results reported in the literature on affective priming (Fazio et al., 1986, Hermans et al., 2001, Hermans et al., 2003, Musch and Klauer, 2003). This explanation implies that FRN positivity can reflect positive affect over and above prediction error computations. In line with this interpretation, it has previously been suggested that the FRN is sensitive to emotional variables (Hajcak and Foti, 2008, Yeung and Sanfey, 2004), and substantial evidence exists demonstrating a relationship between E/FRN amplitude and: (i) affective ratings (Holroyd et al., 2006, Moser and Simons, 2009, Yeung and Sanfey, 2004); (ii) affective traits in healthy participants (Hajcak and Simons, 2002, Hajcak et al., 2003, Luu et al., 2000, Wiswede et al., 2009); and (iii) affective traits in clinical populations (Gehring et al., 2000, Ruchsow et al., 2004, Weinberg et al., 2010).

In summary, four main theoretical models can be derived from the existing literature on the FRN component. First, the original RL-ERN — which suggests that the FRN is a signal of -RPE. Second, valence-independent accounts such as the PRO model, which posit that the FRN is an index of prediction error regardless of outcome valence. The third and fourth models are driven by data showing that the FRN is preferentially modulated by positive rather than negative outcomes in FRN effects. The reward positivity model (or updated RL-ERN) suggests that FRN positivity increases reflect a +RPE signal, whilst a positive affective priming account predicts that the FRN response to rewards should be more positive when positive affective systems have been previously activated.

**Fig. 1:**
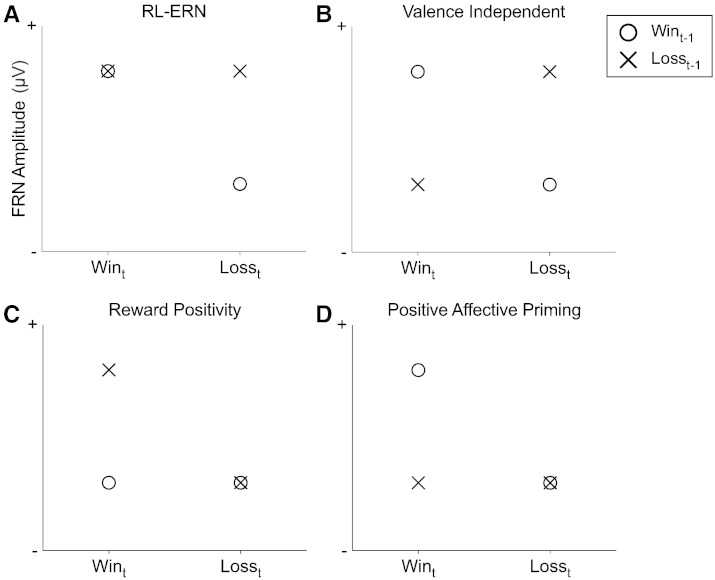
Four predictions of FRN modulation: schematic representation of stFRN encoding predictions in a decision task with randomised outcomes. Abscissa represents Outcome_t_ and ordinate represents relative FRN amplitude — where lower values indicate greater negativity. Circle and cross indicate Win and Loss at Outcome_t-1_, respectively. (A) RL-ERN proposes greatest negativity for losses at Outcome_t_ following win trials at Outcome_t-1_; (B) valence independent models suggest that amplitude negativity should be largest when outcomes deviate from the preceding trial, regardless of outcome valence; (C) the reward positivity model postulates that the FRN is more positive in response to +RPE, i.e. when outcomes are better than expected. (D) A positive affective priming explanation predicts the component will be most positive when positive affective systems have been previously activated i.e. successive Win outcomes.

The goal of the present study was to evaluate whether any of these models could explain the specific case of short-term effects on the FRN (stFRN), defined as the sensitivity of the component to information presented very briefly prior to the decision outcome time-locked to the FRN. In order to address this, we asked participants to perform a multi-trial gambling task where the outcome could be either monetary wins or losses relative to an initial endowment. We separated FRN activity for current trials (Outcome_t_) according to their valence (Win vs. Loss) and according to the valence of the immediately preceding two outcomes (Outcome_t-1_ and Outcome_t-2_). Crucially, the sequence of gains and losses was fully randomised in such a way that participants could only learn that on every trial there is an equal probability of obtaining a reward or a non-reward. This allowed us to target the stFRN by minimising effects due to long-term learning of sequences of outcomes. This procedure also allowed us to formulate distinct predictions for each of the four accounts described above, illustrated in Fig. 1.

## Materials and methods

### Participants

Twenty-nine right-handed (Edinburgh Handedness Inventory > 40; Oldfield, 1971) healthy participants (17 females; mean age = 20.86 years; SD = 2.5 years; range = 18–30 years) with normal or corrected-to-normal vision and no history of psychiatric or neurological conditions participated in this experiment. Two participants were excluded due to excessive EEG artifacts leading to less than 16 artifact-free trials for at least one of the relevant experimental conditions. All analyses were performed on the resulting sample of 27 participants (17 females, mean age = 20.93 years, SD = 2.59 years, range = 18–30 years). To increase ecological validity, participants were told they would be remunerated based on their performance, but due to the randomised nature of outcomes, all subjects received a fixed amount of £7.50. Participants signed an informed consent document, were fully debriefed and the study was approved by the Ethics Committee in the School of Psychology at the University of Leeds.

### Procedure and design

**Fig. 2:**
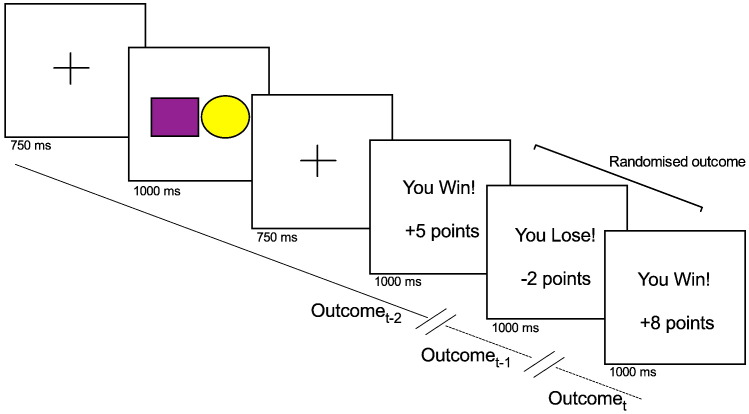
Decision task: each trial started with a fixation cross (750 ms), followed by the presentation of a screen including a circle and a square (one shape coloured yellow and the other purple) representing risky and safe choices. Choice selection was made with a stimulus response pad indicating a decision by pressing the left button for choices that appeared on the left side of the screen and pressing the right buttton for options on the right side of the screen. Next, another fixation screen was displayed, followed by a screen providing a feedback (1000 ms) detailing whether participant had won or lost points in the trial (see “Experimental task” for more details). Feedback outcome was fully randomised on each trial and analysis focussed on current trial outcome (Outcome_t_) and feedback from the preceding two trials (Outcome_t-1_ and Outcome_t-2_).

The experiment took place in a quiet room with lights dimmed. After the setup of the EEG electrode net, participants were invited to sit comfortably at approximately 50 cm away from a computer screen and were instructed to position their right hand on a stimulus response pad (Psychology Software Tools Serial Response Box, Pittsburgh, PA). The experiment was displayed on a 17″ Dell monitor, with a screen resolution of 1280 × 1024 (refresh rate 60 Hz) and controlled by E-Prime® v1.2 (Psychology Software Tools, Pittsburgh, PA). The task closely followed the procedure outlined in Mushtaq et al. (2013). Prior to the experiment, participants were told that they would be taking part in a gambling game in which they should choose a combination of “risky” and “safe” choices across trials to try to maximise the amount of points won and that this amount would be translated into an actual sum of money of up to £10. The verbatim instructions relative to this aspect of the task were as follows: “You must use a combination of ‘risky’ and ‘safe’ choices throughout the task in order to maximise your score”. No reference to rules or sequence learning was provided. As explained previously (see Mushtaq et al., 2013), this approach enabled us to control for the type of behavioural choice preceding the outcome, a variable that can sometimes modulate the FRN (Schuermann et al., 2012) and is often not explicitly controlled. On each trial (see Fig. 2), participants were first shown a fixation cross for 750 ms, followed by a screen displaying two shapes; a circle and square (1500 ms), with one shape coloured yellow and the other purple (each shape 11 cm × 10.5 cm). For half the participants, yellow coloured shapes were classed as “risky” options and purple shapes were “safe” and this rule was reversed for the other half of participants. The association between coloured shapes and response type (risky vs. safe) was counterbalanced across participants. Choosing a risky option would lead to a relatively large amount of points (a randomised amount between 5 and 9 points) gained or lost, whereas a safe choice would lead to a relatively low amount (a randomised amount of points between 1 and 4) of points won or lost. As soon as the coloured shapes appeared on screen, participants had to choose between these two options with a key press. In order to minimise strategic no-responses, if no key was pressed 1500 ms after the onset of the screen presenting coloured shapes, a randomised amount between 1 and 9 points was deducted from the total score. After choice selection, a fixation cross (750 ms) preceded the feedback presentation stimuli, which appeared on the screen for 1000 ms. The feedback screen provided information about the valence of the feedback (“You Win!” or “You Lose!”), a plus or minus signal to indicate reward or punishment and the amount of points to be added or subtracted from the total score. Importantly, both outcomes were equally weighted i.e. the probability of receiving a reward and punishment was identical (i.e. 50%). The outcome on each individual trial (reward vs. punishment) was determined using the software randomisation function built in to E-Prime® v1.2. Across subjects, this produced a comparable number of trials for each outcome sequence combination: Win_t-2_Win_t-1_Win_t_, M = 50, SD = 9; Win_t-2_Win_t-1_Loss_t_, M = 51, SD = 4; Win_t-2_Loss_t-1_Win_t_, M = 52, SD = 6; Win_t-2_Loss_t-1_Loss_t_, M = 53, SD = 4; Loss_t-2_Win_t-1_Win_t_, M = 51, SD = 4; Loss_t-2_Win_t-1_Loss_t_, M = 53, SD = 5; Loss_t-2_Loss_t-1_Win_t_, M = 52, SD = 4; and Loss_t-2_Loss_t-1_Loss_t_, M = 52, SD = 9. Analysis on decision outcomes revealed no significant difference in frequency of win (M = 205, SD = 9) and loss (M = 209, SD = 8) trial outcomes across subjects (t(26) = 1.22, *p* = .232). In order to minimise fatigue, participants were provided with a self-paced break every 40 trials. In total, the task lasted approximately 40 min (including breaks) alongside an additional 25–30 min for technical set up for EEG data acquisition.

### Electrophysiological data recording and analysis

EEG data were recorded with a 128-channel net connected to a high-input amplifier (Electrical Geodesics, Inc., Eugene, OR; for electrode montage see Fig. 1 in Mushtaq et al. Data in Brief, Section 1) at a rate of 500 Hz (0.01–200 Hz bandwidth), and an impedance ≤ 20 kΩ for frontocentral electrodes. Data were recorded using a Cz reference online and digitally converted to an average mastoids reference offline. The ERP module of BESA 5.1 (MEGIS Software GmbH, Gräfelfing, Germany) was used for analysis. Following inspection of raw data, bad channels were replaced using a spherical spline interpolation method implemented in BESA. EEG data were further filtered offline (0.1–30 Hz bandwidth) and segmented into epochs of 0–1000 ms time-locked to the onset of feedback presentation (with an additional 200 ms pre-feedback baseline). Eye movement artifacts were corrected using a multiple source analysis method (Berg and Scherg, 1994, Ille et al., 2002) as implemented in BESA 5.1 (“surrogate method”). In addition, for each channel, epochs with a difference between the maximum and minimum voltage amplitude > 120 μV and a maximum difference between two adjacent voltage points > 75 μV were rejected (after eye movement artifact correction). Participants with less than 16 artifact-free trials in any relevant condition were excluded from the sample (see section Participants). On average, 43 artifact-free trials per condition were accepted for the Outcome_t-2_ × Outcome_t-1_ × Outcome_t_ design.

### ERP quantification

Quantifying the FRN can often be problematic as it overlaps with two other decision-related components — the P2 and P3. This is particularly troublesome when these components are also sensitive to task-related parameters e.g. outcome magnitude and valence. To ensure the robustness of the FRN results reported here, three steps, widely practiced in the literature, were taken:

(1) In our primary analysis, we quantified the FRN as a peak-to-peak difference in the P2–N2 complex (Frank et al., 2005, Holroyd et al., 2006, Moser and Simons, 2009) in order to isolate ERPs related to reward and punishment on outcomes at each trial. Specifically, peak-to-peak amplitudes were computed by subtracting the maximal positive peak in the 150–250 ms time window from the maximal negative peak in the 250–350 ms window. This approach is used because absolute FRN values (i.e. negative peak or mean voltage amplitudes) can be biased by amplitude differences in the preceding P2 — a component that shares a similar scalp topography (Bellebaum et al., 2010b, Ferdinand et al., 2012). This is often the preferred method to disentangle the FRN from other components (Osinsky et al., 2012).
(2) In secondary analyses, we also isolated the component using a difference waveform approach and computed mean amplitude between 228 and 344 ms — an interval identified in a recent meta-analysis of the literature (Sambrook and Goslin, 2015).^1^ This analysis provided comparable results to our peak-to-peak measures and therefore, for conciseness, we present these results in Mushtaq et al. Data in Brief, Sections 2, 3, 4 & 6.
(3) We present differences between relevant conditions at each electrode site through presentation of topographical maps. Consistent with previous research (Walsh and Anderson, 2012), we anticipated that the FRN should show a frontocentral topography.

In addition to the above, we also adopted a region of interest approach to statistical analysis of the FRN. As the component is observed primarily in midline frontocentral sites (Holroyd et al., 2004, Walsh and Anderson, 2012, Yeung and Sanfey, 2004), we formed a cluster in which we averaged electrode data from a group of midline electrodes surrounding the standard FCz location (EGI electrode numbers: ‘12’, ‘5’, ‘6’, ‘13’, ‘112’, ‘7’, ‘106’, ‘Cz’, ‘31’, ‘80’ and ‘55’). Pooling single electrode data in clusters improves the stability of ERP data, attenuates familywise statistical errors (Oken and Chiappa, 1986) and is consistent with common practice in high-density EEG research (Bland and Schaefer, 2011, Walker et al., 2011, Watts et al., 2014). In order to ensure that our results were not due to the utilisation of a cluster of electrodes rather than single electrodes, we verified that similar results were obtained using a single electrode approach. We subjected amplitude measures from electrode e6, which approximates the FCz standard location, to the same analysis for the FRN and e76, which approximates Pz, for the feedback-related P3. There were no differences in the pattern of results for clustered and individual electrode analysis. Therefore, for conciseness, clustered electrode data results are reported. Where clustered effects reached statistical significance, single electrode data also reached significance (*p* < .05), unless otherwise stated.

In addition to analysis on the FRN, we also focused on the feedback-related P3 — a component that has previously been shown to be sensitive to outcome processing (Gu et al., 2011, Yeung and Sanfey, 2004). We did not have a priori hypotheses regarding the feedback-related P3, but we examined it in order to allow comparisons with previous research. As this component is usually measured in posterior sites (Wu and Zhou, 2009), we created a midline parietal cluster surrounding the standard Pz location (EGI electrode numbers: ‘61’, ‘78’,‘62’, ‘67’, ‘72’, ‘77’, ‘71’ and ‘76’). Given that amplitude differences at the onset of the P3 (around the N200) were visible, we calculated both peak-to-peak and mean amplitude measures. Peak-to-peak amplitudes were obtained by subtracting negative peak amplitudes from a 250–350 ms time window from the positive peak amplitude obtained from the 350–500 ms time window. It has to be noted that the P3 (or P300) that is typically reported in feedback monitoring experiments (here, we label this the “feedback-related P3”) conforms to the “P3b” — a subtype of the P300 usually observed in posterior sites (Polich, 2007). There is an additional subtype of the P300 — the P3a, which has a topography that overlaps with the FRN. We also analysed it in order to check if the FRN was clearly differentiated from the P300. To this end, the P3a peak-to-peak amplitude in the frontocentral electrode cluster was examined. We found qualitatively different patterns of results for the FRN and P3a demonstrating the independence of these measures. P3a results are reported in Mushtaq et al. Data in Brief, Sections 4.2, 5 & 6.2.

For the FRN and the feedback-related P3, ERP amplitudes were separated for current outcome (Outcome_t_ = Win_t_ or Loss_t_) by the outcomes of the preceding two trials (Outcome_t-1_ and Outcome_t-2_). We separately considered the effects of choice (“Risky” vs. “Safe”) but found that this factor did not modulate the relationship — see Mushtaq et al. Data in Brief, Section 6. Therefore, for conciseness, and given our focus on the effects of prior and current outcomes on the FRN, we decided to target only Outcome factors in our analyses. Specifically, we predicted that if the FRN for the current trial (Outcome_t_) is influenced by the outcome of the preceding trial (Outcome_t-1_), we should observe an Outcome_t_ × Outcome_t-1_ interaction. Otherwise, we should observe only a main effect of Outcome_t_ with no interaction. In addition, following the suggestion of an anonymous reviewer, we examined difference waveforms for “Stay” trials (in which the behavioural choice was identical to the previous trial) and “Switch” trials (in which the behavioural choice was different compared to the previous trial). In all analyses, significant interaction terms were followed up by post-hoc simple effect analyses, and we considered statistical effects to be reliable at *p* ≤ .05.

## Results

### Behavioural results

**Fig. 3:**
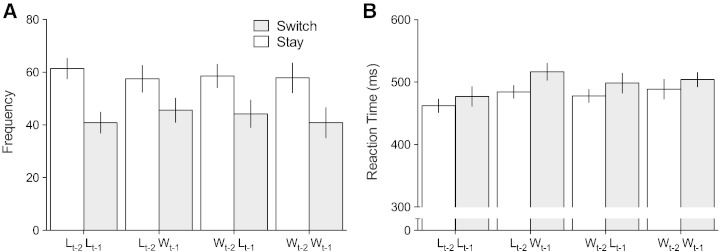
Switch and Stay responses separated by Outcomes_t_ and Outcomes_t-1_: (A) participants showed a bias towards selecting the same choice consecutively over switching — an effect that was not modulated by feedback; (B) response times were faster on trials where a choice was congruent with the previously selected decision. Error bars represent 95% CIs.

Given that behavioural responses (risky vs. safe choices) were not predictive of feedback outcome, the type of choice preceding feedback cannot be considered a meaningful behavioural correlate of the FRN. Nevertheless, in order to assess whether the previous two outcomes influenced the risk propensity, we analysed response frequency using an Outcome_t-2_ × Outcome_t-1_ × Choice_t_ (Risk vs. Safe) repeated measures ANOVA. We found no effects of Choice_t_ (F [1, 26] = 0.14, *p* = .71, *η*^2^*_p_* = .005) — in other words, there was no significant difference between the number of Risk (M = 200.00, SD = 45.55) and Safe choices (206.85, SD = 49.54) and no interactions related to prior outcomes reached significance (F's < 2.38, *p*'s > .135). Next, we analysed the behavioural data by collapsing across risk and safe choice and separating trials for choices congruent with the previous selection (a Stay response) and incongruent (a Switch response). An Outcome_t-2_ × Outcome_t-1_ × Choice_t_ (Stay vs. Switch) ANOVA revealed a significant main effect of Choice (F [1, 26] = 36.95, *p* < .0001, *η*^2^*_p_* = .587), with Stay responses more frequent (M = 235.34, SD = 27.7) than Switch (171.52, SD = 27.58; see Fig. 3A). No interactions between response and prior outcomes reached significance (F's < 2.3, *p*'s > .142).

We also examined the effects of prior outcomes on reaction time (RT) data with an Outcome_t-2_ × Outcome_t-1_ × Choice_t_ repeated measures ANOVA separately for Risk vs. Safe Choices and Stay vs. Switch responses. For the Risk vs. Safe comparison, there were no differences in RTs related to Choice_t_ (F [1, 26] = 0.1, *p* = .760, *η*^2^*_p_* = .004; Risk M = 484.19 ms, SD = 95 ms, Safe M = 487.26 ms, SD = 90 ms) and Choice did not interact with prior outcomes (F's < 1.76, *p*'s > .196). Consistent with a large body of evidence demonstrating that negative affective experiences lead to faster behavioural responses (Hermans et al., 2001, Murphy and Zajonc, 1993, Ohman et al., 2001), we found RTs were facilitated by prior loss outcomes. This effect was observed in Outcome_t-2_ (F [1, 26] = 6.12, *p* = .02, *η*^2^*_p_* = .191, Loss_t-2_ M = 481.56 ms, SD = 92 ms, Win_t-2_ M = 488.49 ms, SD = 93 ms), and strongest in Outcome_t-1_ (F [1, 26] = 21.33, *p* < .0001, *η*^2^*_p_* = .451; Loss_t-1_ M = 475.28 ms, SD = 91 ms, Win_t-1_ M = 494.95 ms, SD = 96 ms). No interactions related to Outcome_t_ and Choice_t_ reached significance (F's < 1.76, *p*'s > .19). For Switch/Stay RT analysis, in addition to the aforementioned facilitation of response time by prior negative outcomes, we also found a significant main effect of Choice (F [1, 26] = 11.36, *p* = .002, *η*^2^*_p_* = .304; Switch M = 499.02 ms, SD = 92 ms, Stay 478.29 ms, SD = 95 ms), but no interactions with prior outcomes (F's < 2.04, *p*'s > .16).

### Electrophysiological results

#### FRN

**Fig. 4:**
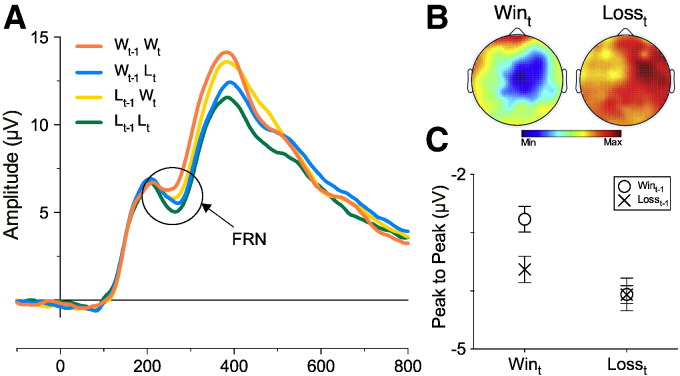
Current FRN amplitude varies as a function of prior outcome: (A) ERP waveforms from the midline frontocentral cluster separated for Outcome_t_ (labelled W_t_ for current Win and L_t_ for current Loss trials) and Outcome_t-1_ (W_t-1_ for prior Win and L_t-1_ for prior loss trial). Abcissa represents time in milliseconds; (B) Topographical maps display peak-to-peak FRN difference between Loss_t-1_ and Win_t-1_ for Win_t_ and Loss_t_ outcomes across the scalp (0 μV to − 3.2 μV); (C) Outcome_t_ × Outcome_t-1_ interaction plot with values taken from the peak-to-peak FRN amplitude. Error bars represent ± 1 S.E.M.

Consistent with previous findings, we found a significant main effect of Outcome_t_ (F[1, 26] = 6.16, *p* = .02, *η*^2^*_p_* = .192), with larger peak negativity for Loss_t_ (M = − 4.06 μV, SE = .76 μV) relative to Win_t_ (M = − 3.2 μV, SE = . 62 μV) trials. Main effects of Outcome_t-1_ (F [1, 26] = 3.87, *p* = .06, *η*^2^*_p_* = .13) and Outcome_t-2_ (F [1, 26] = 3.3, *p* = .079, *η*^2^*_p_* = .114) approached, but did not reach significance. A significant Outcome_t_ × Outcome_t-1_ interaction (F [1, 26] = 5.23, *p* = .031, *η*^2^*_p_* = .167; see Fig. 4) was observed. Subsidiary analyses revealed that the contrast between Win_t-1_ (M = − 2.77 μV, SE = .61 μV) and Loss_t-1_ (M = − 3.63 μV, SE = .67 μV) was significant for Win_t_ (F [1, 26] = 9.03, *p* = .0058, *η*^2^*_p_* = .258) but there was no difference between Win_t-1_ (M = − 4.06 μV, SE = .79 μV) and Loss_t-1_ (M = − 4.06 μV, SE = .75 μV) for Loss_t_ trials (F [1, 26] < 0.01, *p* = .99, *η*^2^*_p_* < .001), indicating short-term changes are encoded only in the processing of Win-related stimuli, in a manner predicted by a positive affective modulation interpretation (Fig. 4C). There were also no significant interactions related to Outcome_t-2_ (F's < 0.21, *p*'s > .162). Analysis of these data using difference waveforms confirmed the finding that FRN variation was driven by win-related ERPs (see Mushtaq et al. Data in Brief, Section 2). Typically, expectancy effects on the FRN are quantified by comparing the difference waveform for “expectedness” (calculated by subtracting expected reward ERPs from expected non-reward) with the difference waveform for “unexpectedness” (calculated by subtracting unexpected reward ERPs from unexpected punishment; Holroyd and Krigolson, 2007; Sambrook and Goslin, 2015). Analogous comparisons (difference between Win_t-1_Win_t_ and Loss_t-1_Loss_t_ was contrasted with the difference between Loss_t-1_Win_t_ trials from Win_t-1_Loss_t_ trials; see Mushtaq et al. Data in Brief, Section 3 for details) confirmed the FRN effects were not driven by “unexpected” outcomes.

#### Feedback-related P3

**Fig. 5:**
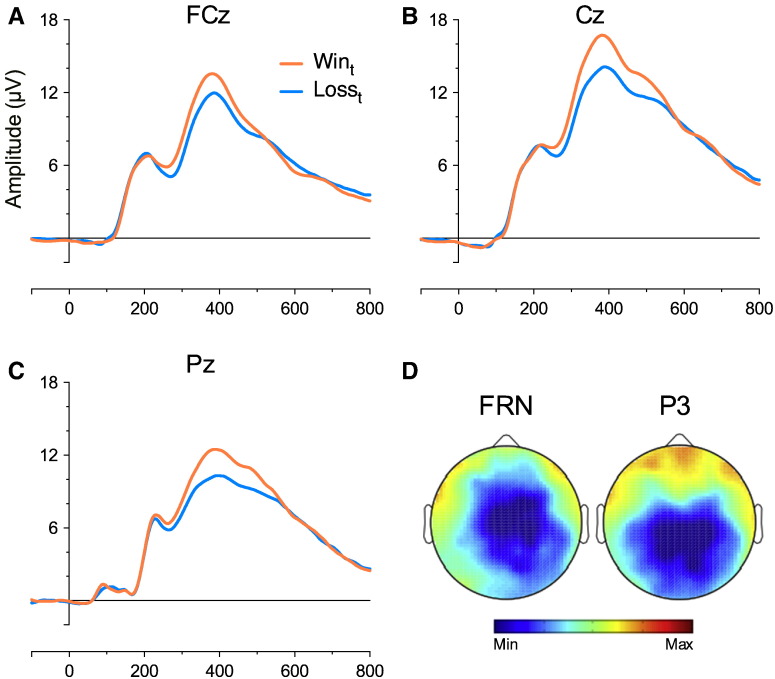
ERPs for Outcome_t_: ERP waveforms from the FCz (A), Cz (B) and Pz (C) separated for Outcome_t._ Abcissa represents time in milliseconds; (D) Topographical map displays mean difference between Loss_t_ and Win_t_ across the scalp for the FRN (0.26 μV to − 1.34 μV) and P3 (0.0 μV to − 2.1 μV).

For the feedback-related P3 we found a significant main effect of Outcome_t_ (F [1, 26] = 13.682, *p* = .001, *η*^2^*_p_* = .345) (see Fig. 5), with a larger peak for Win_t_ outcomes (M = 9.07 μV, SE = .95 μV) relative to Loss_t_ outcomes (M = 7.6 μV, SE = .9 μV). Visualising the scalp maps (Fig. 5B) revealed the effect was posteriorly distributed, with a topography commensurate with that of the classic P3b (Polich, 2007). No other main effects or interactions reached or approached significance (F's < 0.52, *p* values > .51). We also took a mean amplitude measure of the P300, which revealed a significant main effect of Outcome_t_ (F [1, 26] = 30.04, *p* < .0001, *η*^2^*_p_* = .536) with a larger mean for Win_t_ outcomes (M = 13.0 μV, SE = 1.17 μV) relative to Loss_t_ outcomes (M = 10.8 μV, SE = .95 μV). The Outcome_t-1_ × Outcome_t-2_ interaction approached significance (F [1, 26] = 3.61 *p* = .069, *η*^2^*_p_* = .122), but decomposing this revealed no statistically reliable effects (*p* values > .12). These findings are consistent with previous research, as the majority of existing studies report a larger P3 peak for positive compared to negative feedback (Deng et al., 2012, Zhou et al., 2010), although this effect can reverse in environments in which losses are more infrequent than gains (Bland and Schaefer, 2011, Mushtaq et al., 2013).

### Switch vs. stay expectancy

Whilst the pattern of the FRN did not interact with the type of choice selected (i.e. Risk vs. Safe; see Mushtaq et al. Data in Brief, Section 6), reward expectancy might have been contingent on response outcome combinations of previous trials,^2^ and in particular if participants on any given trial chose the same type of gamble as in the previous trial (“Stay” response), or a different type of gamble (“Switch” response). Previous research (e.g. Holroyd and Coles, 2002) indicates that the repetition of similar choices across trials reflects the existence of strong predictions about future outcomes, whereas exploring different behavioural choices might reflect a feeling of uncertainty about future outcomes (Behrens et al., 2007, Mushtaq et al., 2011). Consequently, it could be hypothesised that, if only Stay trials are considered, then FRN results compatible with RL should be observed — given that Stay trials would reflect the existence of stable predictions. To examine these possibilities, Outcome_t1_ × Outcome_t_ ANOVAs were conducted separately for Switch and Stay response trials for the FRN and P3 (P3a peak-to-peak results reported in Mushtaq et al. Data in Brief, Section 5.2). In addition, difference waves for Expectancy (Expected Outcome difference vs. Unexpected Outcome difference — calculated in the same manner described above) were also separated by Response (Stay vs. Switch) and results from these analyses are reported in Mushtaq et al. (Data in Brief, Section 4).

#### FRN: Switch vs. Stay

For Stay trials, we found a significant main effect of Outcome_t_ (F [1, 26] = 11.49, *p* = .0022, *η*^2^*_p_* = .306), with Loss_t_ (M = − 4.31 μV, SE = .74 μV) more negative than Win_t_ (M = − 3.21 μV, SE = .63 μV), but no effect of Outcome_t-1_ (F [1, 26] = .33, *p* = .571, *η*^2^*_p_* = .012) or an Outcome_t_ × Outcome_t-1_ interaction (F [1, 26] = .007, *p* = .933, *η*^2^*_p_* < .001). For Switch trials, the main effect of Outcome_t_ (F [1, 26] = 7.04, *p* = .013, *η*^2^*_p_* = .213) was significant with Loss_t_ (M = − 4.64 μV, SE = .81 μV) more negative going than Win_t_ (M = − 3.59 μV, SE = .62 μV) but there was no effect of Outcome_t-1_ (F [1, 26] = 1.44, *p* = .241, *η*^2^*_p_* = .053). These results were qualified by a significant Outcome_t_ × Outcome_t-1_ interaction (F [1, 26] = 5.46, *p* = .027, *η*^2^*_p_* = .174). Decomposing the interaction revealed a significant effect of Outcome_t-1_ on Win_t_ trials (F [1, 26] = 8.22, *p* = .008, *η*^2^*_p_* = .24), with Loss_t-1_-Win_t_ more negative (M = − 4.21 μV, SE = .74 μV) than Win_t-1_-Win_t_ (M = − 2.98 μV, SE = .55 μV), but there were no difference in Loss_t_ trials (F [1, 26] = 1.16, *p* = .29, *η*^2^*_p_* = .043). None of the predictions derived from RL models (see Figs. 1A–C) are confirmed by the FRN data in Stay trials. However, the results obtained in Switch trials are compatible with the predictions set out in Fig. 1D and are also comparable to the pattern of results obtained when all trials were included in the analysis. Insofar as Switch trials may reflect a heightened perception of uncertainty, then these results could suggest that an affective modulation of the FRN is largest when uncertainty is high.

These effects raised a further question of whether the type of switch response influenced FRN activity. For example, it is possible that a Risk_t-1_-Safe_t_ switch might reflect different outcome expectations to a Safe_t-1_-Risk_t_ response sequence. Subsequent analysis demonstrated that the direction of switch did not modulate activity at Outcome_t_ (Mushtaq et al. Data in Brief, Section 7).

#### Feedback-related P3: Switch vs. Stay

We found an Outcome_t-1_ × Outcome_t_ interaction (F [1, 26] = 6.15, *p* = .002, *η*^2^*_p_* = .191; Fig. 7C) for Switch responses (but not Stay, F [1, 26] = 2.41, *p* = .133, *η*^2^*_p_* = .085; Fig. 7D). This measure showed a main effect of Outcome_t-1_ in Win_t_ (F [1, 26] = 14.35, *p* = .0008, *η*^2^*_p_* = .356) with consecutive Win_t-1_ leading to increased positivity (M = 9.38 μV, SE = .99 μV) relative to Loss_t-1_ (M = 7.56 μV, SE = .72 μV). There was also a large effect of Outcome_t-1_ in Loss_t_ (F [1, 26] = 41.92, *p* < .0001, *η*^2^*_p_* = .617), with Win_t-1_ leading to a larger positivity (M = 7.76 μV, SE = .72 μV) than Loss_t-1_ (M = 4.79 μV, SE = .47 μV).

## Discussion

The goal of this manuscript was to evaluate which of four theoretical accounts of the FRN could best explain short-term FRN effects (stFRN). In a multi-trial gambling task in which the valence of outcomes was fully randomised, we observed that the reward positivity amplitude to “win” trials increased when it was immediately preceded by a single win outcome compared to when it was preceded by a loss outcome (at Outcome_t-1_). The results from this study are inconsistent with models that solely relate the FRN to prediction error computations; however, they are compatible with an affective-motivational model of the FRN. We discuss the implications of these results below.

The results of this study are clearly incompatible with predictions derived from RL-based models (see Figs. 1A–C). However, they match the predictions laid out in Fig. 1D — which suggests that these data could be explained by an effect of positive affective priming. A reward at Outcome_t-1_ could have primed a positive affect system thus driving the results observed here. This prior activation could have made this system more sensitive to incoming positive stimuli, thus facilitating the response to positive feedback at Outcome_t_. This is consistent with a body of research showing that the ability to be primed is an important feature of affective systems (Fazio, 2001, Fazio et al., 1986, Hermans et al., 2001, Hermans et al., 2003, King and Schaefer, 2011, Lang et al., 1990, Sabatinelli et al., 2001). It is worth noting that whilst the literature on affective priming indicates that both positive and negative affective systems are sensitive to priming, the present data show that only FRN waveforms to “Win” trials were sensitive to Outcome_t-1_. These findings are consistent with recent studies (e.g. San Martín et al., 2010, Mushtaq et al., 2013) showing that FRN to win outcomes is enhanced in contexts where positive outcomes are expected, whereas FRNs to negative outcomes do not vary significantly. These findings are also more generally consistent with the view that the component is mostly sensitive to positive rather than negative outcomes (Deng et al., 2012, Mushtaq et al., 2013, Sambrook et al., 2012, San Martín et al., 2010). Moreover, these results are in line with the idea that positive and negative affect systems can function independently (Lang and Bradley, 2010), which is supported by data from fMRI studies demonstrating that rewards and punishments are processed via dissociable neural circuitry (Elliott et al., 2000, Elliott et al., 2003, Liu et al., 2007, Nieuwenhuis et al., 2005, O'Doherty et al., 2001). It is also important to note that an affective explanation of the FRN has been proposed previously (Hajcak and Foti, 2008, Luu et al., 2000, Yeung and Sanfey, 2004) and further research will be needed to explore the possibility that the FRN is a specific index of positive affective states.

Despite the apparent contradiction between these results and RL-derived predictions, it is important to stress that our results do not invalidate the idea that the FRN is sensitive to quantitative and subjective RPEs. Indeed, there is substantial evidence in the literature to affirm this possibility (Walsh and Anderson, 2012) and it is most likely that this is the primary factor influencing FRN modulation. However, the current results point to an additional factor that determines the FRN. Our data, combined with existing evidence in the literature, lead us to propose the possibility that the FRN is determined by prediction errors only when stable prediction can be formed (which is typically achieved by learning from non-random sequences of outcomes). Otherwise, if there is a state of uncertainty driven by the impossibility of forming strong outcome predictions, then the FRN is determined by affective systems. Existing research on the FRN when the formation of predictions is inhibited has shown that the FRN effect can be observed when learning and expectation formation is restricted (Ma et al., 2012) (although see Bismark et al. (2013) for different results). These findings, alongside the current results suggest that prediction error is not the sole determinant of FRN variation.

**Fig. 6:**
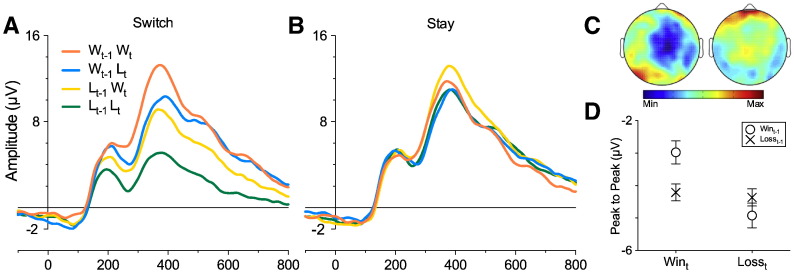
Switch vs. Stay FRN: ERPs from the midline frontocentral cluster separated for Outcome_t-1_ and Outcome_t_ in Switch (A) and Stay (B) responses. Abcissa represents time in milliseconds; (C) topographical maps display FRN difference for Loss_t-1_ and Win_t-1_ during Win_t_ (left) and Loss_t_ (right) outcomes across the scalp (0.7 μV to − 2.4 μV); (D) Outcome_t_ × Outcome_t-1_ interaction for Switch responses using a peak-to-peak FRN amplitude. Error bars represent ± 1 S.E.M.

**Fig. 7:**
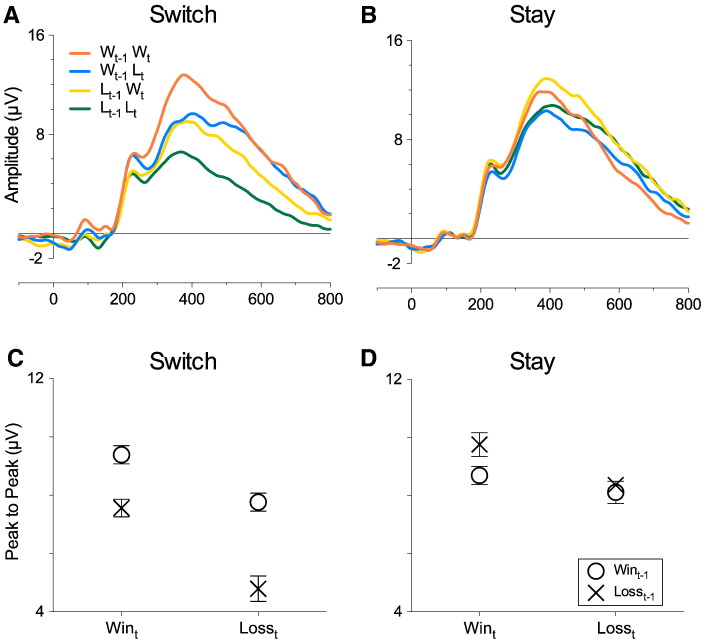
Switch vs. Stay P3: ERPs from the midline parietal cluster of electrodes separated by Outcome_t_ and Outcome_t-1_ for (A) Switch and (B) Stay trials. Abcissa represents time in milliseconds. Peak-to-peak measures demonstrate the relationship between Outcome_t-1_ and Outcome_t_ for Switch (C) and Stay (D) responses.

The suggestion that the FRN is modulated by affective factors when predictions are weak or non-existent is also supported by the results obtained when we separated the data by Switch and Stay trials. Our results show very clearly that, in Stay trials, FRN effects are relatively weak (see Fig. 6B) and do not conform to any of the four models presented in Fig. 1. In Switch trials, we observed exactly the same pattern of results observed when all trials (Switch and Stay) were included in the analysis (and consistent with Fig. 1D). This finding has two implications: First, the initial hypothesis that reinforcement learning processes would be at play for Stay trials is disconfirmed; Second, the pattern of results compatible with an affective modulation of the FRN is specific to Switch trials. It is plausible that Switch trials reflect a heightened perception of outcome uncertainty, as it has been suggested that diversifying behavioural choices is typical of contexts where predictions are weak or non-existent (Behrens et al., 2007, Mushtaq et al., 2011, Sutton and Barto, 1998). Therefore, these results may suggest that outcome monitoring systems are determined by affective processes if levels of perceived uncertainty are high. This explanation leads to another consequence: Although preventing the formation of stable predictions is enough to block RL-based outcome monitoring, the influence of affective systems on outcome processing would also be conditional to a heightened perception of uncertainty. More research will be needed to explore these possibilities, in which levels of uncertainty are explicitly manipulated in contexts where prediction formation is suppressed.

Beyond the debate on the determinants of FRN effects, it is also important to consider previous studies that have examined the effects of prior outcomes on the FRN. Overall, these studies have yielded contradictory results. For instance, Osinsky et al. (2012) found that the largest negativity in FRN amplitude was associated with activity at Outcome_t_ that was opposite to the outcomes from the previous two trials — a finding that is consistent with valence-independent models and in particular with the PRO model (Alexander and Brown, 2010, Alexander and Brown, 2011). However, these authors used a pseudo-randomisation approach that led to the systematic manipulation of the repetitions of similar outcomes at Outcome_t-2_ and Outcome_t-1_, in order to create expectations influencing ERPs at Outcome_t_. Therefore, learning of expectations may have been allowed to develop in their study even though it involved only a recent outcome history. In the present study, the full randomisation of outcome valence at trial level appears to have annulled any potential effect of prior outcomes at t_- 2_, indicating that the effects observed here do not reflect expectations built over sequences of trials.

In a seminal study, Gehring and Willoughby (2002) also examined the effects of previous outcomes on the FRN. These authors reported that the magnitude of the FRN (characterised as the difference between amplitude related to positive and negative feedback) increased when preceded by a loss — seemingly contradictory to the results observed in the present study. Although the utilisation of a difference waveform in that study does not allow a test of the four models evaluated here, the results do seem to differ from ours in that negative and not positive feedback at Outcome_t-1_ influenced the FRN. However, in that experiment, previous losses seemed to be associated with a greater probability of making a risky choice on the current trial. It is therefore possible that the association between previous losses and a larger FRN magnitude reported in that study reflected in part that individuals with greater risk propensity have a strong subjective expectation of gains. In other words, the manipulation of the riskiness of choices during the experiment may have facilitated the generation of predictions on a trial-by-trial basis. This would imply that subjective perceptions of risk could potentially cause mechanisms similar to RPE computations on a very short time scale.

Our findings seem to contradict to the data reported by Holroyd and Coles (2002), who demonstrated that ERN activity was modulated in consecutive choice-outcome combination trials on an Eriksen Flanker Task (EFT) in a manner consistent with RL-ERN. However, our main pattern of results was obtained in “Switch” trials, which are conceptually opposite to the consecutive-choice trials employed by Holroyd and Coles (2002). When only “Stay” trials were considered in our study, we observed a pattern compatible with the findings of Holroyd and Coles (2002), albeit not statistically significant (see Mushtaq et al. Data in Brief, Fig. 4). A number of additional factors could have contributed to differences between these studies. For example, the task requirements were fundamentally different — in the EFT, subjects were required to identify whether a central stimulus (letter) was compatible/congruent with surrounding stimuli (sequence of letters surrounding the central stimulus — flankers). In our task, subjects had to decide which of the two shapes on the screen (one representing a risky choice and the other a safe option) would be the most appropriate choice to maximise the amount of points gained over the course of the task. Thus, motivation and task objectives fundamentally differed between these two experiments, which makes comparisons between these studies relatively difficult. Future research will be needed to explore if these factors can have a differential impact on FRNs relative to switch or stay trials.

In addition to the FRN, we also analysed the feedback-related P3. We did not have specific hypotheses about this component but we analysed it to allow comparisons with previous research. Results are consistent with existing studies in that P3 amplitudes were more positive for Win than Loss feedback — which is compatible with the idea of a preferential attentional processing of positive outcomes (e.g. Gable and Harmon-Jones, 2007, Harmon-Jones and Gable, 2009). Switch vs. Stay analyses also revealed interesting additional results showing that, for Switch trials, the P3 for both Win_t_ and Loss_t_ was enhanced if a Win was obtained in the previous trial (Win_t-1_). The larger positivity for positive compared to negative outcomes is usually seen as a preferential attentional processing for positive outcomes, and some authors suggest that this could also reflect positive prediction error, which is possible when stable predictions can be formed (Pfabigan et al., 2010). In the current study, the fact that Win_t-1_Win_t_ gave rise to a more positive P3 than Loss_t-1_Win_t_ contradicts positive prediction error explanations. Furthermore, the general influence of Win_t-1_ could be interpreted as a priming of affect-related attentional processes (Bradley, 2009, Bradley et al., 2001, Gable and Harmon-Jones, 2007, Lang et al., 1997). We speculate that if an enhanced attentional response is triggered by a Win feedback, it is plausible that attentional systems will retain a heightened level of readiness long enough to facilitate attentional processing of the next feedback, regardless of valence. Against this hypothesis, one could argue that the larger magnitude of the effect of prior outcomes on Loss_t_ trials compared to Win_t_ trials could reflect the action of a positive prediction error mechanism. However, as stated previously, this interpretation is ruled out by the observation that Win_t-1_Win_t_ is linked to a more positive P3 than Loss_t-1_Win_t_. A possible explanation for this difference in magnitude of the effects of prior positive outcomes is that P3 positivity could have reached a ceiling effect in Win_t-1_Win_t_, thus reducing the magnitude of the effect between positive current outcomes. Ceiling/floor effects can be observed in brain activity (Büchel et al., 2002) and in the P3 more specifically (Gonsalvez and Polich, 2002). Given that we did not have a priori hypotheses for this component, these interpretations need to be taken with caution and more research will be needed to establish the robustness of these effects.

Finally, it is important to discuss three potential limitations of this study: First, our findings could potentially be explained by a subjective +RPE account (i.e. the elicitation of a +RPE when outcomes were subjectively better than expected). The reasoning behind this account is that winning on several consecutive trials in a row in a randomised outcome scenario could be interpreted as positive surprise. If this hypothesis held true, it could be predicted that the surprise would be present — and even greater — if participants experienced three wins in a row. However, our data show no effects of Outcome_t-2_ on FRN amplitude and thus do not support this hypothesis. Instead, these data point towards an FRN modulation that is driven by a distinctly different process to that of objective and subjective prediction error. Second, as mentioned previously, we do not exclude the possibility that potential ceiling or floor effects may have played a role in our P3 data (and this cannot be excluded for the FRN data either), although the possibility of such effects cannot by themselves explain our main findings. Such factors are seldom considered in ERP research (however, see Gonsalvez and Polich, 2002), and future studies should consider to what extent these potential effects can modulate the P3 and FRN. Third, research on the FRN (and other neural signals) often tends to contrast distinct theoretical models focused on unique potential mechanisms. An issue to consider in future FRN research is whether different models could co-exist. For instance, in situations where long-term predictions can be formed, it is plausible that different groups of neurons involved in outcome monitoring would be dedicated to distinct mechanisms. From this perspective, it could be speculated that certain neurons would be more sensitive to prediction errors — following behaviour consistent with reinforcement learning models, and other neurons would be more sensitive to affective parameters, and both groups would have a joint influence on FRN amplitude. It is also possible to posit that certain neurons would be sensitive to positive prediction errors, and others to a generic “surprise” mechanism as defined by the PRO model — indeed, studies have demonstrated the existence of such neurons in primates (Hayden et al., 2011, So and Stuphorn, 2012). By consequence, future research will need to consider that the FRN can be modulated by multiple determinants.

### Conclusions

In summary, we obtained results suggesting that the FRN is primarily modulated by affective factors when incapacity to form strong predictions causes uncertainty. Importantly, these data indicate that a multifactorial explanation of the nature of the FRN is necessary — one that must consider affective and motivational factors in outcome processing in addition to prediction error.

## Conflict of interest

The authors declare no competing financial interests.

## Footnotes

1 This time-window was suggested by an anonymous reviewer. We note that the same pattern of results was obtained using the 250–350 ms time window adopted in the primary analysis.

2 We thank an anonymous reviewer for this suggestion.
